# The SAIL MELD-B e-cohort (SMC) and SAIL MELD-B Young adult e-cohort (SMYC) – a reproducible methodological pipeline for dynamic cohort curation and research

**DOI:** 10.23889/ijpds.v9i5.2753

**Published:** 2024-09-18

**Authors:** Roberta Chiovoloni, Nisreen Alwan, Ann Berrington, Michael Boniface, Jakub Dylag, Nic Fair, Simon DS Fraser, Emilia Holland, Rhiannon K Owen, Sebastian Stannard, Zlatko Zlatev, Ashley Akbari

**Affiliations:** 1 Population Data Science, Swansea University Medical School, Faculty of Medicine, Health and Life Science; 2 School of Primary Care, Population Sciences and Medical Education, Faculty of Medicine, Southampton General Hospital; 3 Department of Social Statistics and Demography, University of Southampton; 4 School of Electronics and Computer Science, University of Southampton

## Objectives

Through the Multidisciplinary Ecosystem to study Lifecourse Determinants and Prevention of Early-onset Burdensome Multimorbidity (MELD-B) project, we established the SAIL MELD-B e-cohort (SMC) and the SAIL MELD-B Young adults e-cohort (SMYC), with the aim to enhance the understanding of ‘burdensomeness’ in individuals living with multimorbidity, including identifying new clusters of burdensome indicators, exploring early life risk factors and modelling potential preventative scenarios.

## Method

We use routinely-collected anonymised linked demographic, health and administrative data sources available within the SAIL Databank to define SMC and SMYC. These cohorts were developed using a reproducible, maintainable, methodological pipeline that allows for dynamic updates as data coverage expands. The pipeline efficiently processes new burdensomeness concepts, facilitating the extraction of relevant records associated with the concepts identified for use in the SMC and SMYC.

## Results

SMC and SMYC comprises of 5,180,602 and 896,155 individuals registered with a Welsh General Practice at any time between 1st January 2000 and 31st December 2022 respectively. Analysis of primary and secondary care health data reveals that the most common conditions in SMC were depression (21.6%), anxiety (21.1%), asthma (17.5%), hypertension (16.2%), and atopic eczema (14.1%). In SMYC, the most common conditions were atopic eczema (21.2%), asthma (11.6%), anxiety (6.0%), deafness (4.6%), and depression (4.3%).

## Conclusions and Implications

SMC and SMYC provide two generalisable population samples, which can be used to address various research questions across MELD-B. The adaptability of the methodological pipeline allows cohort curation to be repurposed for other projects accessing population-scale data sources and trusted research environments.

